# Comparison of cannabidiol to citalopram in targeting fear memory in female mice

**DOI:** 10.1186/s42238-020-00055-9

**Published:** 2020-12-11

**Authors:** Zackary T. Montoya, Amy L. Uhernik, Jeffrey P. Smith

**Affiliations:** Colorado State University-Pueblo, 2200 Bonforte Blvd, Pueblo, CO 81001 USA

**Keywords:** Fear learning and memory, Cannabidiol (CBD), Citalopram, Selective serotonin reuptake inhibitor (SSRI), Extinction, Female, Sexual dimorphism

## Abstract

**Background:**

Cannabidiol (CBD) and selective serotonin reuptake inhibitors (SSRIs) are currently used to treat post-traumatic stress disorder (PTSD). However, these drugs are commonly studied after dosing just prior to extinction training, and there are gaps in our understanding of how they affect fear memory formation, their comparative effects on various types of memory, and of sexual dimorphisms in effects. Also, more studies involving female subjects are needed to balance the gender-inequality in the literature. Therefore, the purpose of this study was to directly compare the effects of CBD to citalopram in affecting the formation of auditory cued, contextual, and generalized fear memory, and to evaluate how extinction of these different memories was altered by pre-acquisition treatment in female mice. We also evaluated the impact of the estrous cycle on each of these.

**Methods:**

Auditory-cued trace fear conditioning was conducted shortly after dosing female C57BL/6 mice, with either CBD or citalopram (10 mg/kg each), by pairing auditory tones with mild foot shocks. Auditory-cued, contextual, and generalized fear memory was assessed by measuring freezing responses, with an automated fear conditioning system, 24 h after conditioning. Each memory type was then evaluated every 24 h, over a 4-day period in total, to create an extinction profile. Freezing outcomes were statistically compared by ANOVA with Tukey HSD post hoc analysis, *N* = 12 mice per experimental group. Evaluation of sexual dimorphism was by comparison to historical data from male mice.

**Results:**

Auditory cue-associated fear memory was not affected with CBD or citalopram; however, contextual memory was reduced with CBD by 11%, *p* < 0.05, but not citalopram, and generalized fear memory was reduced with CBD and citalopram, 20% and 22%, respectively, *p* < 0.05. Extinction learning was enhanced with CBD and citalopram, but, there was considerable memory-type variability between drug effects, with freezing levels reduced at the end of training by 9 to 17% for CBD, and 10 to 12% with citalopram. The estrous cycle did not affect any outcomes.

**Conclusions:**

Both drugs are potent modifiers of fear memory formation; however, there is considerable divergence in their targeting of different memory types which, overall, could support the use of CBD as an alternative to SSRIs for treating PTSD in females, but not males. A limitation of the study was that it compared data from experiments done at different times to evaluate sexual dimorphism. Overall, this suggests that more research is necessary to guide any therapeutic approach involving CBD.

## Introduction

Behavioral fear-memory experimentation in rodents and other animals evaluates multiple discreet forms of learning and memory to model particular human psychological disorders. For example, tone-associated fear memory experimentation in rodents is a model for auditory processing disorder, while contextual, and generalized fear memory experimentation models post-traumatic stress disorder (PTSD). Despite the clinical relevance, research into female-specific attributes of fear learning and memory are historically underrepresented in behavioral neuroscience (Ramikie and Ressler 2018; Bergstrom 2016; Barha and Galea 2010; Daskalakis et al. 2013; Choleris et al. 2018; Zhao et al. 2018). This has limited development of treatment approaches specifically tailored for effective care for women. The importance of generating gender-specific products of learning and memory research is underscored by more recent reports demonstrating a significant degree of sexual dimorphism in fear learning and memory, and related disorders. For example, despite men experiencing greater levels of exposure to trauma, the prevalence of PTSD in women is twice that of men, and women experience more severe symptoms (Merz et al. 2018; Clark et al. 2019; Inslicht et al. 2013; Chen et al. 2014; Farrell et al. 2013). While this would suggest that sexual maturity and the associated variation in sex hormone signaling might play a role in these sexual dimorphisms, the literature is conflicting, and it is not clear whether hormonal fluctuations in the estrous cycle influence the prevalence and severity of PTSD in females (Cossio et al. 2016; Zhao et al. 2018; Kirry et al. 2019; Graham and Scott 2018; Maeng et al. 2017; Graham and Daher 2016; Kobayashi et al. 2020; Maddox et al. 2018; Day and Stevenson 2020; Day et al. 2016). Therefore, there is a need for additional fear learning and memory studies, which involve female subjects.

PTSD is a disorder of learning and memory characterized by the generalization of fear memory and associated behavioral responses to inappropriate stimuli (Chen et al. 2014; Atwoli et al. 2015). PTSD affects over 350 million people worldwide, with an individual lifetime prevalence of 7.3% (Koenen et al. 2017; Hoppen and Morina 2019; Karatzias et al. 2018). Leading clinical therapies for PTSD include cognitive behavioral therapy, eye movement desensitization therapy, and reprocessing therapy. However, these approaches are minimally effective and consequently are often used in combination with pharmacotherapeutics (Gallagher 2017; Simpson et al. 2019; Garcia et al. 2019). This prominently includes the use of selective serotonin reuptake inhibitors (SSRIs) which were originally developed for depression. However, SSRIs produce gender-dependent variability in effects, can initially increase negative symptoms, require chronic treatment, are problematic to discontinue, and can produce severe negative side effects including depression, violent behavior, and suicide (Burghardt et al. 2004; Burghardt and Bauer 2013; Tawa and Murphy 2013; Soga et al. 2012; Soga et al. 2010; Clayton et al. 2006; Bezchlibnyk-Butler et al. 2000; Molero et al. 2015; Surawski and Quinn 2011; Healy et al. 2006). Thus, there is a need to develop alternative therapeutics for PTSD which would include the benefits of SSRIs without the harmful side-effects.

Cannabidiol (CBD) is currently being promoted as one such alternative candidate, with published evidence that it exerts anxiolytic properties which promote extinction of fear memories (Bitencourt et al. 2008; Loflin et al. 2017; Campos et al. 2016; Stern et al. 2018). CBD is reported to have low toxicity and is well tolerated in humans, although the volume of clinical research into its efficacy for treating PTSD is currently minimal (Iffland and Grotenhermen 2017). Despite the reported potential for CBD as therapeutic for PTSD, we recently showed that a single dose administered just prior to fear memory acquisition enhances the expression of fear in male mice, consistent with other research showing an anxiogenic effect when administered to rats (Uhernik et al. 2018; ElBatsh et al. 2012). This would suggest a counter indication for CBD as a treatment for PTSD. Moreover, very little work has evaluated the effects of CBD on fear learning and memory in females (Shbiro et al. 2019), and no work has directly compared the effects of CBD to SSRIs on fear learning and memory. It would, however, be very useful to know how CBD and SSRIs compare in affecting particular types of fear memory that model PTSD and other memory disorders; i.e., do these drugs affect multiple memory types the same way, or do differences exist that might guide safe and efficacious prescription for particular disorders but not others?

With this in mind, we designed a behavioral fear learning and memory study using female C57BL/6 mice to directly compare the effects of CBD and the SSRI, citalopram, on auditory-cued, contextual, generalized fear, and extinction fear learning and memory. We interleaved the drug treatments in these experiments, with drugs acutely administered prior to trace fear conditioning, such that our results could be directly compared with our previously published results in male mice (Uhernik et al. 2018). Trace fear conditioning was used because it requires processing in brain centers that control both cognitive and autonomic behaviors, and therefore is an excellent paradigm for studying disorders of learning and memory, such as PTSD, which also involve cognitive and reflexive processing (Dunmore et al. 1999; Meiser-Stedman 2002; Ferreri et al. 2011; Han et al. 2003). Finally, because sexual dimorphic learning and memory traits are widely attributed to sex hormone-dependent processes, we evaluated the impact of the estrogen- versus progesterone-dominated phases of the estrus cycle on the behavioral outcomes. Our results show that CBD produces similar, but broader, effects on fear memory than does citalopram, and provides evidence of gender-divergent effects for CBD. These results will help guide future fear learning and memory research aimed at evaluating the efficacy of these treatments for PTSD and other learning and memory disorders.

## Methods

### Subjects

All experimental procedures were carried out in accordance with approved Colorado State University-Pueblo Institutional Animal Care and Use Committee protocols and guidelines. Female C57BL/6 mice (Charles River Laboratories) arrived at the age of 29 to 32 days old and were housed in groups of three under a 12 h dark-light cycle and given food and water ad libitum. Mice were weighed 24 h after arrival and weights were distributed across experimental groups to ensure similar group averages. All mice were acclimated for 10-18 days prior to the start of experimentation.

### Pharmacological treatments

All solutions were prepared immediately before use. Cannabidiol (CBD) and citalopram (CIT) were dissolved in 2% ethanol, 2% tween-80, and 0.9% saline. The vehicle was identical without the addition of CBD or CIT. All solutions were delivered intraperitoneally (IP) with CBD and CIT administered at 10 mg/kg and an equivalent volume was used for vehicle-treated controls (volumes varied with mouse weight and averaged 307 +/− 16 μl). CBD and vehicle were injected 30 min prior to trace fear conditioning (Uhernik et al. 2018) and CIT was administered 60 min prior (Burghardt et al. 2013), each as previously described.

### Fear conditioning apparatus

All experiments were performed using an automated, computerized fear conditioning chamber as previously described (Uhernik et al. 2018). In brief, the apparatus consisted of two plexiglass chambers, each with a sound-attenuating isolation cubicle equipped with a ventilation fan, a top-mounted USB camera, and a house light mounted on the side wall. Foot shocks and auditory cues were delivered through a removable floor grid by the Actimetrics FreezeFrame software. Freezing responses were captured on digital video and analyzed using motion detection software (Actimetrics).

### Stimuli

The conditioning stimulus (CS) was always an audible 85db, 7 kHz, 30 s tone. The unconditional stimulus (US) was always a 1-s long 0.5 mA foot shock. Both stimuli were computer controlled and delivered by the fear conditioning system described above.

### Context A and context B

All fear conditioning and testing procedures were conducted in a dedicated conditioning room as previously described (Uhernik et al. 2018). In brief, animals were transported from their home cages to the conditioning room in a transfer cage bedded with either wood shavings for habituation, trace conditioning, and context A memory testing or shredded paper for cued memory testing. Context A consisted of a grid shock floor, a white-colored back wall, and the chamber was cleaned using 70% ethanol each time before a mouse was introduced to the chamber. Context B consisted of a perforated stainless-steel non-shock floor, a black colored back wall, and vanilla extract was placed in a weigh boat located on the waste collection pan of the chamber to provide a unique odor. The 409 Lemon Fresh Multi-Surface Cleaner was used to clean the chamber between sessions when the chamber was configured for context B.

### Experimental procedure

#### Day 1: Habituation

All mice were divided into three conditioning groups: paired conditioned, unpaired conditioned, and non-conditioned. All mice, regardless of conditioning group, were individually placed in the fear conditioning chamber, configured in context A, and habituated for 20 min before being returned to their home cages. During habituation, mice in the unpaired group received seven 30 s presentations of the conditioning stimulus. Non-conditioned and paired conditioned mice did not receive tone presentations during the habituation period; however, these mice were habituated for the same duration of time as the unpaired group.

#### Day 2: Trace fear conditioning

All fear conditioning was completed in context A, 24 h after habituation, and on the second day of experimentation. Each of the three conditioning groups was further divided into three treatment groups, vehicle, CBD, or CIT. Mice in the vehicle or CBD groups received an IP injection 30 min prior to conditioning whereas those mice that received CIT were injected 60 min prior to fear conditioning. This resulted in a final total of nine experimental groups containing 12 animals per group. Paired conditioning consisted of a 2 min baseline period followed by seven 30-s long presentations of the CS each paired with an US. A trace interval of 17 s was placed between presentations of the CS and US, and the seven CS-US pairs were separated by an inter-trial interval (ITI) of 2 min. Animals in the unpaired groups received seven presentations of the US at pseudo-random intervals. Non-conditioned animals received seven presentations of the CS with a 2-min ITI. All animals were exposed to the conditioning chamber for the same overall duration regardless of the conditioning group.

#### Day 3: Memory testing

Fear memory was evaluated 24 h after conditioning, on the third day of experimentation, in context B where three 30 s tones were presented with each tone separated by a 60-s ITI. For the cued memory test, freezing was measured during a 3 min baseline period prior to the onset of the first tone (referred to as generalized fear), and 10 s following the conclusion of the three tones (referred to as auditory-cued fear). Contextual memory was tested 4 h later by returning the mice to context A for 5 min and measuring the percent freezing over the entire period.

#### Days 4, 5, and 6: Extinction training

On the fourth, fifth, and sixth days of experimentation, 24, 48, and 72 h after the first day of memory testing, respectively, mice underwent extinction training. During these 3 days, mice underwent the exact same testing protocols as described on day 3 described above.

#### Data analysis and statistics

The freezing response of mice was captured at 60 frames per second and analyzed by the motion detection software (FreezeFrame) to generate a motion index. The motion index was binned into 10-s intervals which were averaged during various epochs. All statistics were performed using Rstudio. Two or three-way ANOVAs and Student’s *t* test were used as described in the text and a Tukey HSD post hoc analysis was performed when appropriate.

#### Vaginal lavage and cell imaging

A micropipette tip containing approximately 0.1-0.2 mL of 0.9% sterile saline solution was inserted 1-2 mm into the vaginal opening with care as further insertion can stimulate the cervix and induce pseudopregnancy. The saline solution was flushed in and out of the opening until the solution was visually cloudy and then one to two drops were placed on a clean microscope slide and coverslipped immediately. The cells were viewed and photographed using a light microscope and images from Goldman et al. (2007); Byers et al. (2012); Gal et al. (2014); and Cora et al. (2015) were used as a reference to determine the mouse’s stage in the estrous cycle. Also, concurrently a digital picture was taken of each mouse’s vaginal area to independently determine the stage of the estrous cycle. If the visual and cytology determination did not match, the cytology data was used over the visual data. Lavages were performed starting 4 days prior to habituation and continued daily until the experiment was complete.

## Results

### Auditory cue-associated fear memory was not affected with CBD or citalopram

**Fig. 1:**
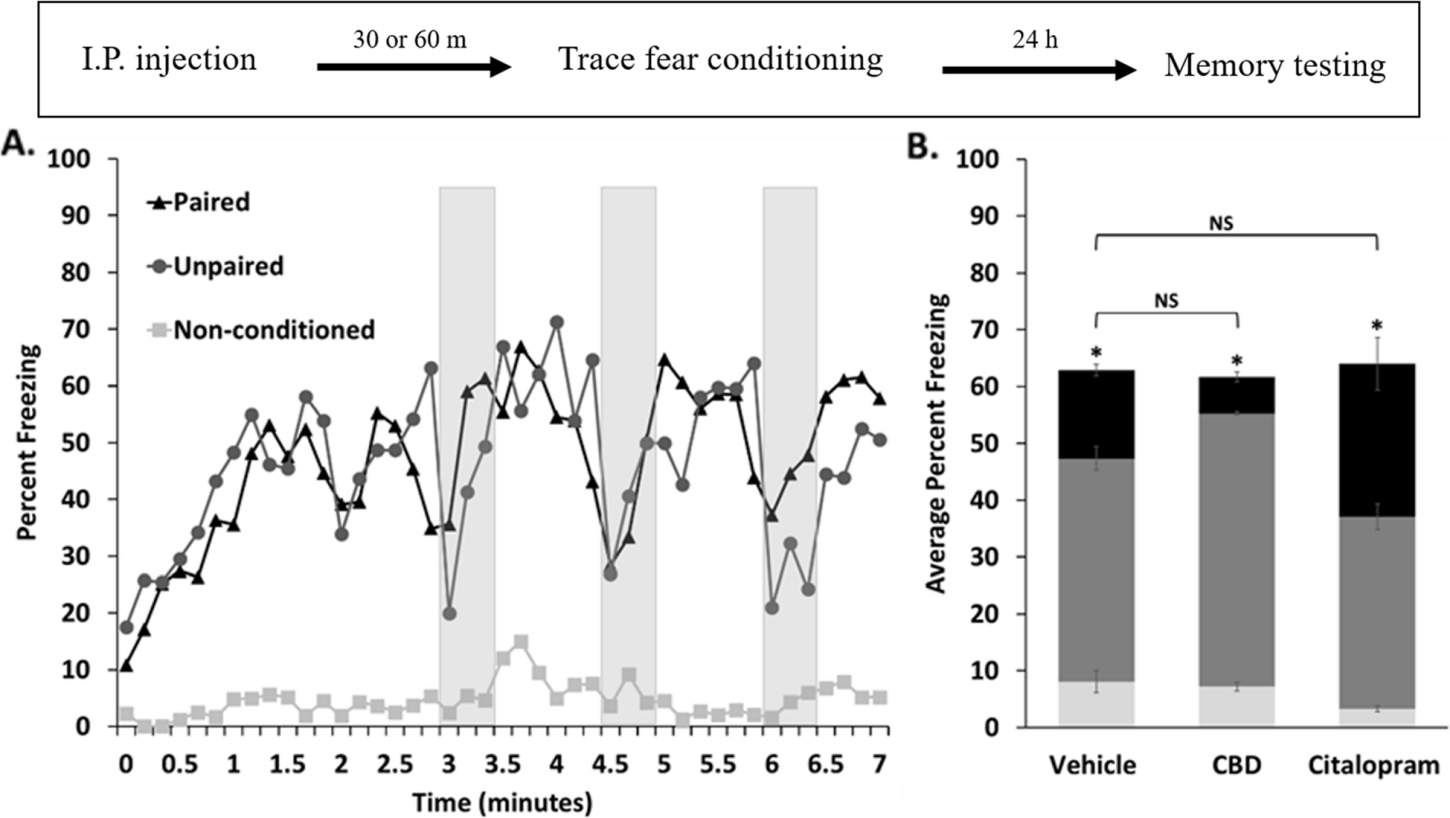
Trace fear-conditioning produced a tone-associated memory in all treatment groups of female mice. **a.** Time course of the average percent freezing during memory testing for vehicle-treated mice that received paired conditioning, unpaired conditioning, and non-conditioning. Shaded bars show periods during which the CS was presented. **b.** Percent freezing was averaged within treatment groups for each conditioning type (black - paired conditioning, dark grey - unpaired conditioning, and light grey - non-conditioned). Auditory-cued memories were validated by statistically comparing freezing levels between mice that received paired versus unpaired conditioning within each treatment group using one-tailed student’s t-tests (*p < 0.05). Comparison of freezing responses between either treatment group that received paired conditioning were not statistically significant (NS) in comparison to control. N = 12 animals per group with standard errors indicated. The timeline for the experiment is shown in the inset above

Adult female C57BL/6 mice were given trace-fear conditioning as described in the materials and methods section. Briefly, 30 or 60 min prior to conditioning, mice received intraperitoneal injections of either vehicle, CBD, or citalopram, respectively, with the time courses and doses chosen so as to be consistent with the previously published literature (Burghardt et al. 2013; Uhernik et al. 2018). Auditory-cued memory was assessed 24 h after conditioning in a novel context by measuring freezing responses to tone presentations and comparing results between mice that received paired versus unpaired conditioning. With this approach, a statistical difference indicates that an auditory-cued memory was present (Fig. 1).

Our results showed very low levels of freezing among mice that received non-conditioning, indicating that treatments did not affect animal mobility in a way that might confound the interpretation that freezing is a measure of memory strength (vehicle vs. CBD *p* = 0.4 ; vehicle vs. CIT *p* = 0.15). On the other hand, mice that received paired or unpaired conditioning showed considerable levels of freezing which increased following the onset of each tone presentation and briefly persisted for a period following the offset of the CS. An auditory-cued memory was shown to be present in all treatment groups by comparing freezing levels between animals that received paired versus unpaired conditioning for each of the three treatment groups. Additionally, mice that received paired conditioning did not show measureable differences in freezing between either treatment group when compared to control. Therefore, neither CBD nor citalopram, when administered prior to memory acquisition, appeared to affect the formation, or recall of the auditory cue-associated memory when assessed 24 h following trace fear conditioning. It was interesting that mice, which received unpaired conditioning showed trends toward increased and decreased freezing behavior with CBD or citalopram, respectively (vehicle vs. CBD *p* = 0.06; vehicle vs. CIT *p* = 0.08). Because this freezing was in a novel context, and to an auditory cue that was not paired with the US during conditioning, it could be interpreted as representing fear generalization to the auditory cue.

### Contextual memory was reduced with CBD but not citalopram

**Fig. 2:**
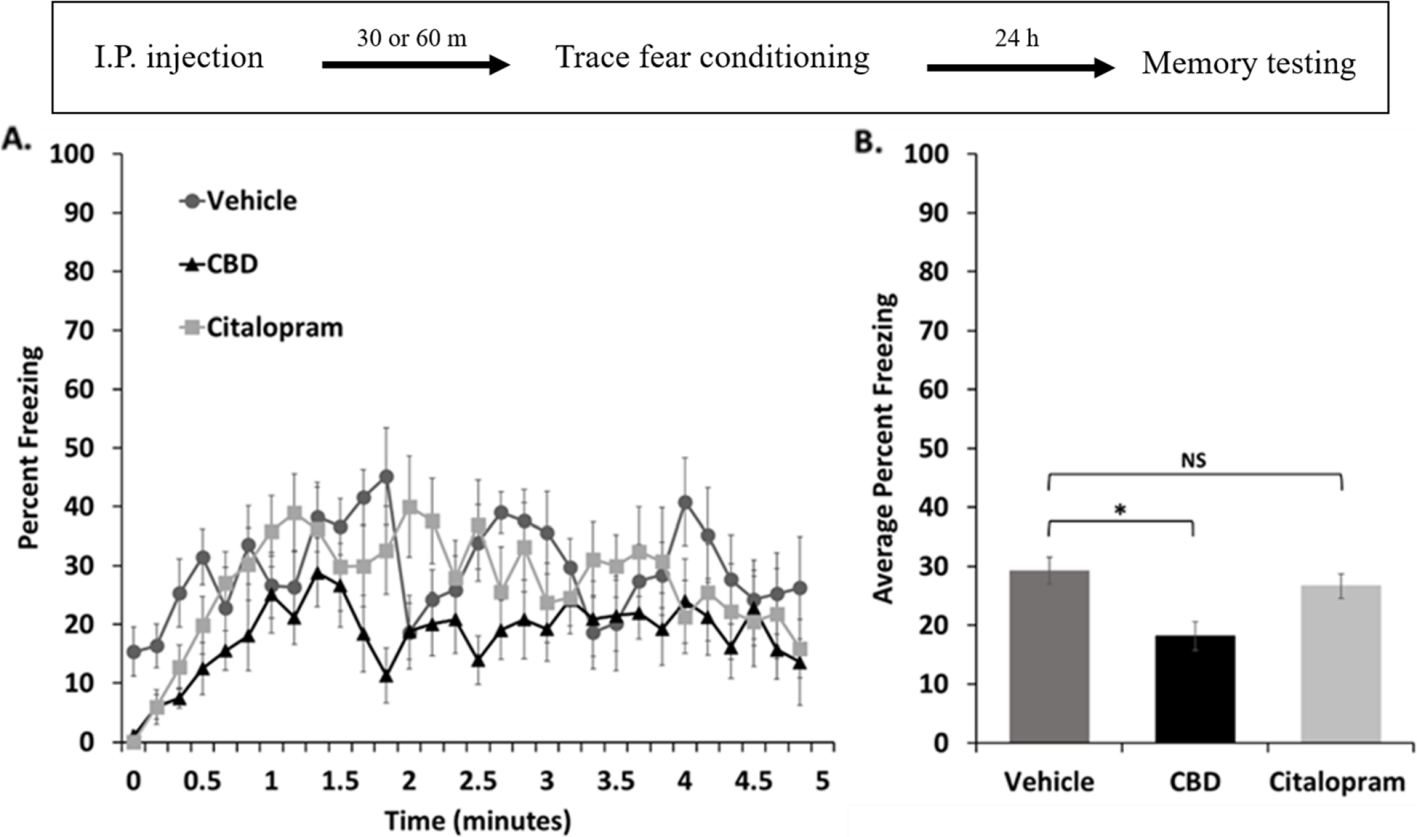
Contextual memory was reduced in female mice with CBD but not citalopram. **a.** Time course of the freezing levels during a five-minute exposure to the original conditioning context for each treatment group. **b.** Freezing levels averaged across the period shown in A with standard errors indicated. Mice that received CBD treatment showed a significant reduction in freezing compared to controls when assessed with a one-tailed t-test (*P< 0.05), however, citalopram treated animals did not show a difference (NS). The timeline for the experiment is shown in the inset above

Four hours following cue-associated memory testing, mice that had received paired conditioning were placed back into the original training context and freezing behavior was averaged across a 5-min period to assess contextual memory (Fig. 2). In this experiment, vehicle-treated mice showed moderate levels of freezing. Mice that had received CBD, on the other hand, showed significantly lower levels of freezing, indicating that treatment inhibited the formation or recall of the context association. In contrast, citalopram treatment did not produce a noticeable change in contextual memory strength.

### Generalized fear memory was reduced with CBD and citalopram

**Fig. 3:**
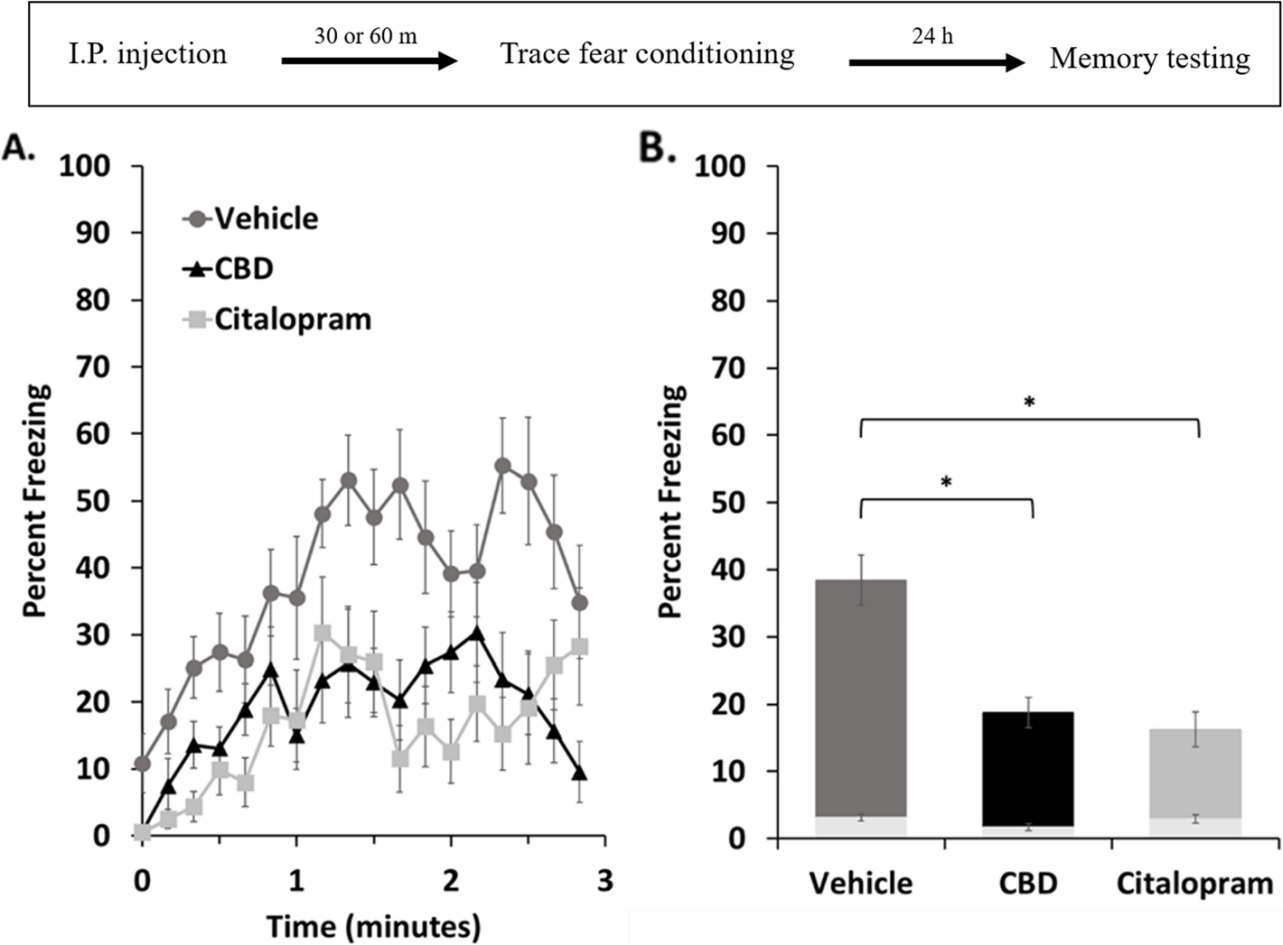
Fear generalization to a novel context was reduced in female mice with both CBD and citalopram. **a.** Freezing levels during a three minute baseline period in the novel context. **b.** Freezing levels were averaged over the entire baseline period and compared between control and each treatment group using one-tailed t-tests (p* < 0.01 with standard errors shown). Freezing among non-conditioned mice is included for comparison (light-shaded portion of bars). The timeline for the experiment is shown in the inset above

Fear generalization to context is commonly measured in a novel context during a baseline period which precedes auditory-cued memory testing with mice that had received paired conditioning. During a 3-min baseline preceding our auditory test, all treatment groups initially showed low levels of freezing which increased during the first minute and then plateaued for the remainder of the test (Fig. 3a). Average freezing levels across this entire period were near 40% for vehicle-treated mice, but dropped to about half of that for CBD and citalopram treated groups (Fig. 3b). This decrease was statistically significant for both treatment groups. Therefore, both CBD and citalopram, when administered prior to memory acquisition, inhibited generalization of fear association with a novel context.

### Extinction learning was enhanced with CBD and citalopram with memory-type variability

Fear memory extinction training was conducted at 24-h intervals for 4 days following the first day of memory testing. It was completed by first exposing mice to the novel context for a 3-min baseline period, followed by seven 30 s presentations of the auditory cue which were spaced at 120 s intervals. In this period, we assessed generalized fear, and auditory cue-associated memory, respectively. Four hours after that, mice were returned to the original context for 5 min to assess contextual memory strength.

**Fig. 4:**
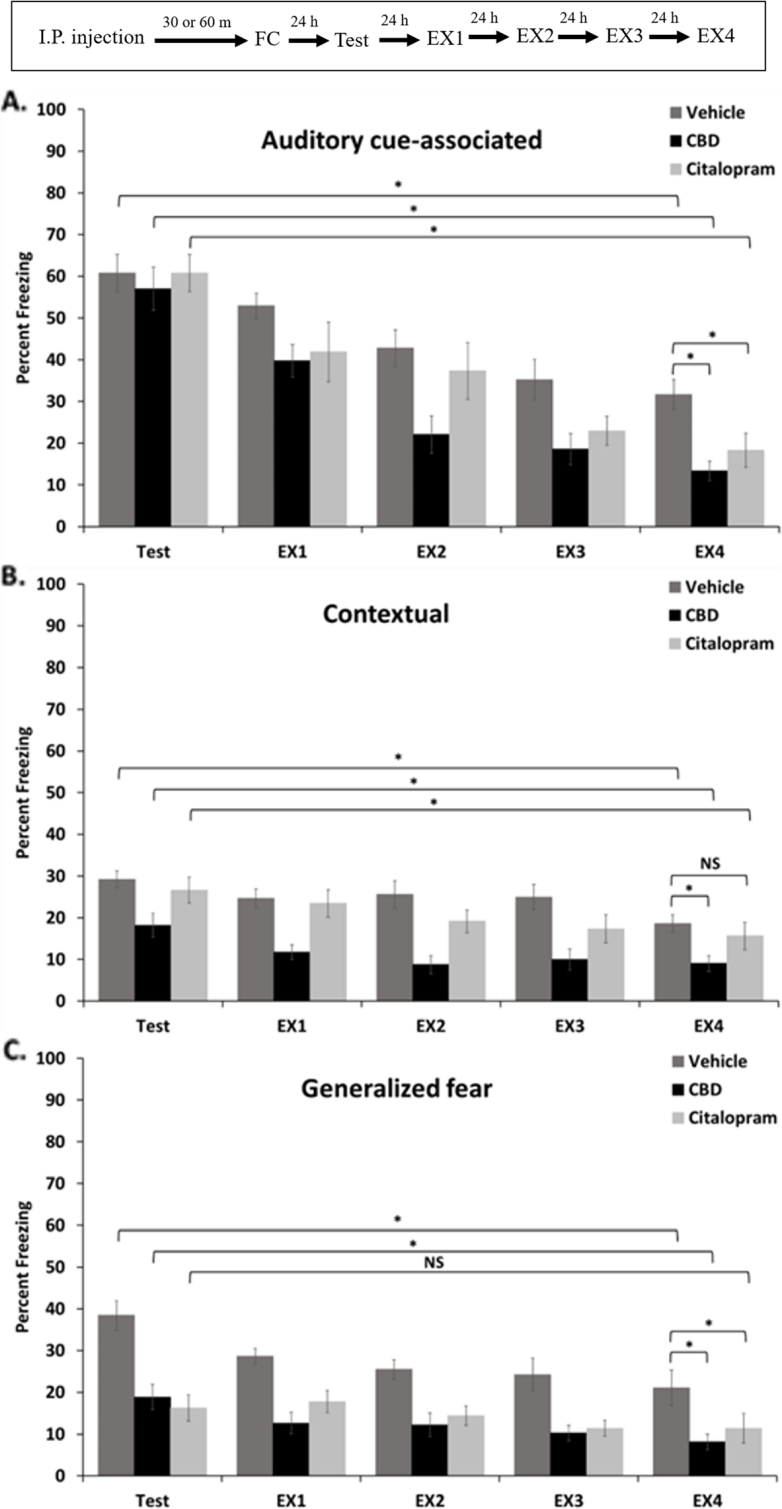
Results of extinction training over a four day period following the original memory test in female mice. **a.** Extinction of the tone-associated memory was assessed in the novel context by recording freezing levels averaged across seven presentations of the auditory cue and comparing these levels within groups across days, and across treatment groups on the last day, with a t-test (*p = < 0.05). **b.**Contextual memory extinction was similarly assessed by averaging freezing levels over a five minute period in the original conditioning context (*p < 0.05). **c.** Extinction of generalized fear was similarly assessed by measuring freezing levels during a three minute baseline period in the novel context (*p < 0.05). The timeline for the experiment is shown in the inset above

For extinction of auditory-cued memory, regardless of treatment, all three groups of mice showed significantly decreased freezing to the auditory cue by the fourth day of extinction training when analyzed within treatment groups across the 5-day period. However, the level of freezing was significantly reduced in both CBD and citalopram treated mice when compared across treatment groups within the final day of extinction training (Fig. 4a). This result shows that extinction learning was present for all groups, and that both CBD and citalopram were able to significantly enhance extinction learning for this memory type, when administered one-time prior to trace-fear conditioning. Notably, the difference in final levels of freezing was not significantly different between the CBD and citalopram treated mice.

Extinction of contextual memory also was significant over the full period of extinction training for each of the treatment groups. However, on the final day of extinction training, CBD-treated mice showed a significantly lower level of freezing in comparison to controls (*p* = 0.001) while citalopram-treated mice were similar to controls (*p* = 0.2), (Fig. 4b). Therefore, CBD significantly enhanced extinction of the contextual memory, but, citalopram did not affect this.

Finally, generalized fear memory was significantly extinguished over the 4 day period of extinction training in both vehicle controls and CBD-treated mice, but not in the citalopram-treated group. Therefore, citalopram-treated animals did not show extinction of generalized fear with our protocol. This was likely a result of the greatly reduced original memory; however, this was also true for the CBD-treated animals which, despite a similar reduction in the memory measured 24 h post-conditioning, did show extinction of the memory (Fig. 4c). In addition, on the final day of extinction training, both CBD- and citalopram-treated mice showed significantly lower levels of freezing in comparison to control (*p* = 0.005 and 0.04, respectively). Therefore, both CBD- and citalopram-treated animals showed reduced levels of fear generalization across the entire experimental period, however, the generalized fear memory was extinguished only in the CBD-, but not the citalopram-treated animals.

### Stages of the estrous cycle did not affect fear memory or its extinction

**Fig. 5:**
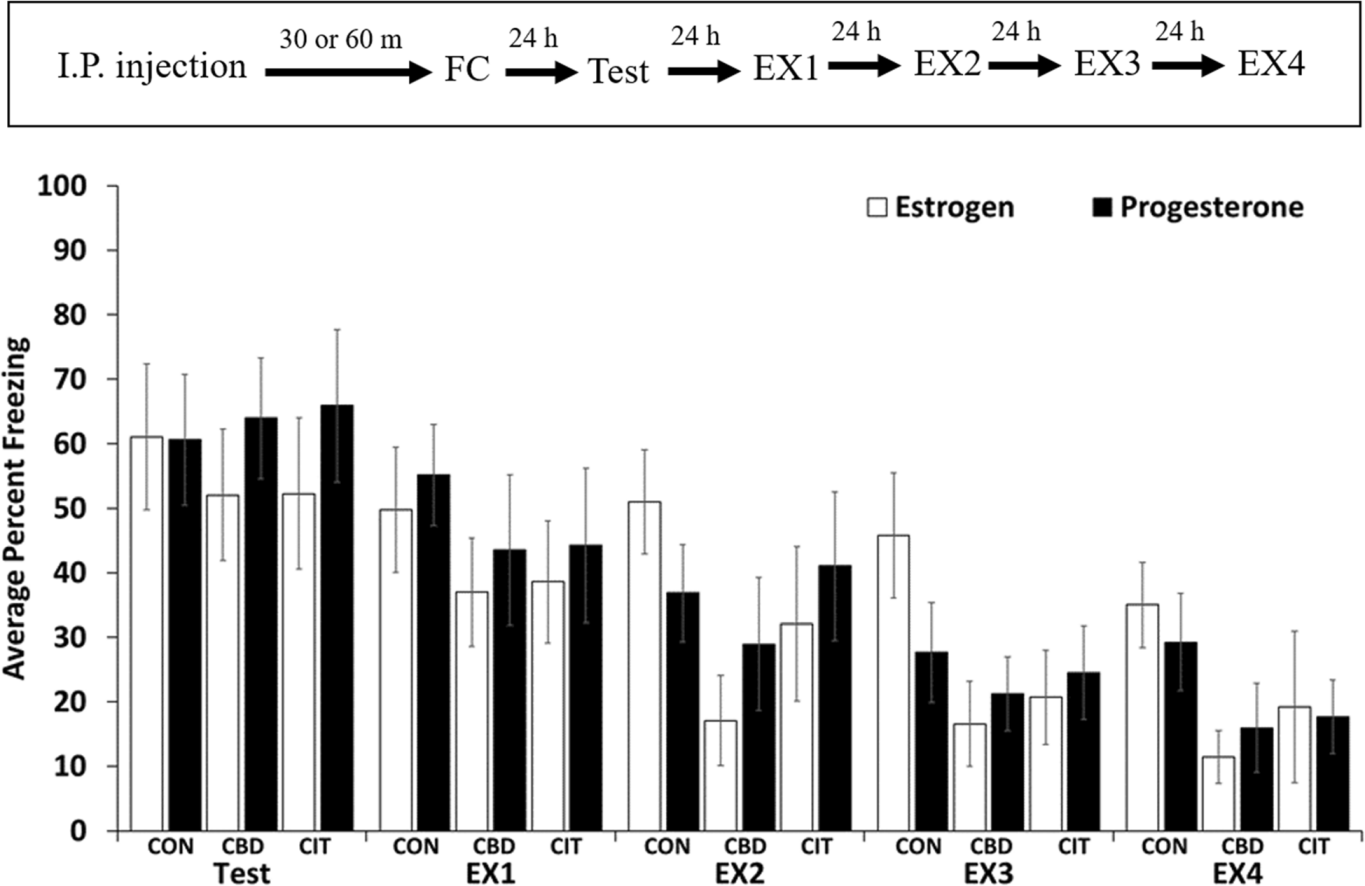
Phases of the estrous cycle did not affect the strength of auditory-cued memory assessed 24 h after conditioning. Results for the other memory types appeared similar (data not shown). Therefore, while certain gender differences were apparent in the effects of CBD and citalopram on fear memory acquisition and extinction, whether female mice were in an estrogen- or progesterone-dominated phase of the sex cycle was not a factor in the experimental outcomes reported in this study. The timeline for the experiment is shown in the inset above

The reported sexually dimorphic effects of CBD and citalopram suggested that the female estrous cycle might impact fear memory recall and extinction in our studies. Therefore, our study design included a step to determine the stage of the estrous cycle, on each day, for each mouse, over the course of experimentation (see materials and methods). Mice were grouped, within treatment groups, into estrogen- and progesterone-dominated phases for each of the types of memory that we assessed: auditory cued, contextual, fear generalization, and extinction of each of these. In no case did we detect a statistically significant impact of the estrous cycle phase on the results presented above. Figure 5 shows the results of this analysis for auditory-cued memory recall.

## Discussion

### Auditory-cued fear memory appeared similar after 24 h but was more easily extinguished with CBD or citalopram

Consistent with our previous study using male mice, a single pre-acquisition dose of CBD to females did not affect auditory cue-associated memory recall when assessed 24 h after trace fear conditioning (Fig. 1) (Uhernik et al. 2018). We saw the same lack of effect using citalopram in this study; however, this is contrary to reports showing an increase in auditory-cued fear following delay conditioning when citalopram was administered with the same timing to male rats (Burghardt et al. 2004; Inoue et al. 1996). This difference could be attributed to differences in processing memories formed with trace versus delay conditioning, the use of rats instead of mice, or to sex differences (Jurkus et al. 2016).

The lack of effect on initial recall was in stark contrast to the marked effect of either drug in enhancing the extinction of the cued memory over the 4-day extinction-training period (Fig. 4), an effect that we also previously observed with CBD using male mice (Uhernik et al. 2018). The pharmacokinetics of CBD suggest that it would by systemically eliminated after 24 h, and therefore, these effects likely resided in the acquisition or early consolidation phase of the original memory, and not with the extinction learning process for CBD. We are the first to evaluate the effects of a single pre-trace-conditioning dose of citalopram on extinction in female mice; however, our result is consistent with other studies showing fear memory extinction-enhancing effects of this drug when applied just prior to extinction training (Inoue et al. 1996; Stahl 1998; Burghardt et al. 2004; Burghardt et al. 2007; Nishikawa et al. 2007; Burghardt and Bauer 2013; Inoue et al. 2014; Bauer 2015). Combined, our results show that the initial memory which formed with trace conditioning after treatment with either CBD or citalopram was comparable in magnitude to that which formed with vehicle-treated controls. However, the extinction data from the same mice suggest that the memory which formed with either drug treatment must in some other way have differed from controls, since these memories were more easily extinguished in both male and female mice. This could be explained by drug-targeting of specific neuronal populations involved in processing the original acquisition in a way that would shift their functional role in the extinction process. For example, intrinsic plasticity in specific populations of prefrontal to amygdala projection neurons is important for extinction of fear memory following trace conditioning, while CBD has previously been shown to affect spine plasticity associated with trace conditioning specifically in the hippocampus, but not the amygdala (Song et al. 2015; Uhernik et al. 2018). Therefore, consistent with the literature, an acute pre-conditioning dose of either drug has the capacity to alter the formation of fear memory, but, in a manner that is not necessarily detectable 24 h after conditioning by a typical memory test. This could have important implications for designing and interpreting fear learning and memory studies involving these drugs and could have possible clinical relevance as will be discussed below.

### CBD causes sex-dependent effects on contextual fear memory and extinction but citalopram does not affect either memory

We previously found that CBD enhances contextual memory and its extinction when assessed 24 h following trace fear conditioning in male mice (Uhernik et al. 2018). In contrast, using an identical experimental design, we found that context-dependent memory and its extinction were decreased by CBD in this study involving female mice (Figs. 2 and 4). There are only three studies where CBD was administered before acquisition of contextual fear memory, two with male rats and one with male mice (ElBatsh et al. 2012; Levin et al. 2012; Uhernik et al. 2018). Interestingly, CBD was anxiogenic in one rat and the mouse study, but anxiolytic in the other rat study, with major differences between the rat studies being the duration of treatment and strain. Therefore, our results suggest a sexual dimorphism in the effect of CBD on contextual memory in mice. This is consistent with well documented sexual dimorphisms in contextual fear memory processing in general (Cossio et al. 2016; Farrell et al. 2013; Jasnow et al. 2006; Kobayashi et al. 2020; Dalla et al. 2009), and noted sexual dimorphisms in the distribution and function of the major receptor signaling pathways that support CBD signaling in rodents (Jimenez Naranjo et al. 2019; Liu et al. 2020; Inoue et al. 2014; Uphouse et al. 1991; Greaves et al. 2005). This result could be important for guiding the design and interpretation of fear memory experiments involving mixed genders and could have clinical relevance as discussed below.

In contrast, we did not see an effect of citalopram on contextual memory or its extinction in this study (Figs. 2 and 4). While a majority of previous studies have shown increased fear and anxiety responses with acute dosing of SSRIs (Burghardt et al. 2004; Burghardt et al. 2007; Ravinder et al. 2011; Mir and Taylor 1997; Spigset 1999; Salchner and Singewald 2002; Sánchez and Meier 1997; Dekeyne et al. 2000), at least one study showed an anxiolytic effect of an acute dose on contextual memory recall, therefore, our result is not inconsistent with the published literature (Inoue et al. 1996). Moreover, we are unaware of any other study that assessed contextual memory recall after administering citalopram to female mice before trace conditioning, therefore, any discrepancy could be related to differences in experimental design and/or to known gender differences in the signaling pathways and brain circuitry that is targeted by citalopram (Berlanga and Flores-Ramos 2006; Burghardt et al. 2013).

### CBD and citalopram inhibited generalized fear and the CBD effect was sexually dimorphic

We previously reported that CBD treatment increased the expression of generalized fear in male mice (Uhernik et al. 2018). In contrast, generalized fear was reduced by CBD and citalopram in this study. This suggested that, like with contextual memory, a sexual dimorphism is present in the effects of CBD on generalized fear memory. Additionally, both drugs significantly decreased the level of generalized fear measured on the fourth day of extinction training relative to control; however, citalopram-treated mice did not show a significant level of extinction when assessed by comparing freezing levels across the 5 days of experimentation (Fig. 4b). This was apparently due to the large reduction in the size of the original freezing response measured as the baseline for extinction 24 h after conditioning. Therefore, the apparent extinction effect of citalopram, and to an extent CBD (which had a similar large effect on the original memory), could be mostly attributable to an inhibition of fear generalization to the novel context, as assessed 24 h after conditioning, and less so to a direct enhancing effect on extinction learning.

Overall, these results are consistent with the majority of published reports which show that both CBD and SSRIs have anxiolytic properties (Bitencourt et al. 2008; Campos et al. 2016; Stern et al. 2018; Homberg 2012). Interestingly, citalopram has previously been shown to be more effective for treating depression in women than in men (Young et al. 2009; Berlanga and Flores-Ramos 2006; Dalla et al. 2010). While we do not have comparable data involving males and citalopram, this sex-dependent difference might suggest that the acute dose we used here might also share with CBD the sexual dimorphism in affecting fear generalization. This would be interesting to evaluate in the future and could support the development of preclinical and clinical studies with this drug, which is known to be underrepresented in studies involving females (Choleris et al. 2018; Tronson 2018).

### Stages of the estrous cycle did not affect fear memory or its extinction

The female mice in our study were 39 to 50 days old when we began fear conditioning. This is past the range in which these mice are known to reach sexual maturity. Therefore, the lack of any estrous phase influences on the drug effects in our study suggest that hard-wired sexual dimorphisms in brain physiology with a developmental basis, rather than acute influences of sex hormone signaling to fear memory, was the source of the gender-dependent divergence in effects that we report here. This is consistent with well documented pre-pubescent gender-specific differentiation of contextual fear processes in rodents and humans, which of importance, are thought to provide a basis for sex differences in anxiety and stress disorders in people. Interestingly, this is also known to begin during sexual differentiation, early in development, of key brain areas that process fear learning and memory, including the hippocampus and amygdala (Colon et al. 2018; Koss and Frick 2017; Fish et al. 2020). On the other hand, while there are still many unknowns regarding acute sex hormone signaling to the fear learning processes which we studied with mice, there is some related research showing hormonal effects, which involve pro-estrous phase signaling in women. For example, an estradiol-dependent enhancement of brain activity in key areas that support fear memory acquisition and extinction has been shown in women (Velasco et al. 2019; Hwang et al. 2015; Peyrot et al. 2020). Moreover, a majority of relevant studies showing sex-cycle-dependent effects on fear learning and memory involved stress as a factor (Maeng and Milad 2015; Cover et al. 2014; Van Veen et al. 2009; Ter Horst et al. 2009; Antov and Stockhorst 2014; Garrett and Wellman 2009; Maeng et al. 2010). Perhaps the reason we did not observe effects of the estrous cycle in our studies is that we avoided involving stress as a factor in our experiments.

### Pre-clinical relevance of the divergent effects of CBD and citalopram on fear memory

Both drugs in our study inhibited particular types of fear memory when given prior to conditioning, suggesting the mechanisms of action involved either the acquisition or early consolidation phases of fear memory. This would suggest an acute-phase clinical perspective for these drugs in reducing fear memory formation, perhaps as a prophylactic for people with known risk of developing a fear-memory-related disorder such as PTSD. However, while CBD is thought to be mostly metabolized by 24 h, citalopram has a plasma half-life closer to 35 h, suggesting that it could have also targeted the recall process (Deiana et al. 2012; Sangkuhl et al. 2011). Because the drugs were both likely metabolized at the time of memory testing, the pharmacokinetics also suggest that each drug targeted fear memory, rather than exerting anxiolytic effects during memory testing. This would not, however, rule out the possibility of an anxiolytic effect as part of the mechanism which affected memory formation.

In summary, citalopram inhibited recall of generalized fear; however, CBD reduced both this and context fear when measured 24 h after conditioning. This divergence in effects is consistent with the current understanding of the pharmacology of CBD, which includes a broader range of neurological targets prominently including endocannabinoid signaling, G protein-coupled receptor 55 (GPR55), transient receptor potential vanilloid type 1 (TRPV1) channels, and serotonin 5HT1A receptors. In comparison, citalopram is known to primarily target serotonin signaling, and only mildly interacts with other neurotransmitter systems (Hyttel 1982; Preskorn 1997; Stahl 1998). Since PTSD involves both fear generalization and misrepresentation of contextual fear associations, our study suggests that CBD might provide a spectrum of effects that would be more comprehensive than citalopram for targeting processes involved in acquisition of memories that lead to PTSD. More important, CBD is not known to produce any of the negative side effects associated with SSRI’s, which comparatively, might make it more therapeutically desirable (Mir and Taylor 1997; Spigset 1999; Teicher et al. 1990; Fergusson et al. 2005; Ravinder et al. 2011; Salchner and Singewald 2002; Sánchez and Meier 1997). On the other hand, because CBD inhibited the formation of contextual memory, it could be considered detrimental as a blocker of this essential adaptive learning process. Citalopram did not have this effect.

Overall, perhaps the most remarkable effect that we observed were those on extinction learning, given that extinction effects were assessed 5 days after the drugs were applied. This strongly implies that, as discussed above, the original memory and not the extinction process per se was the target of each drug. The specificity of extinction effects was also different between drugs. This is summarized simply in that CBD-enhanced extinction of auditory-cued, contextual, and generalized fear memory, but citalopram was only able to enhance extinction of the auditory-cued memory. This would suggest that CBD could be more favorable than citalopram for treating PTSD since the disorder involves the broader spectrum of memory types that was more completely targeted by CBD. On the other hand, generalized fear with citalopram showed a large decrease at 24 h, but no further extinction, consistent with the known divergence in effects for this drug over the time course ranging from initial administration to long term use (Berlanga and Flores-Ramos 2006; Bigos et al. 2009; Burghardt and Bauer 2013; Burghardt et al. 2013). This suggests citalopram might have a therapeutic application for fear learning and memory disorders involving a tailored acute pre-memory acquisition administration and would have a less effective or different indication involving chronic treatment. Notably, the final difference in freezing levels for auditory-cued extinction was not significantly different between CBD- and citalopram-treated mice, despite the divergent pharmacology described above.

Finally, our research shows gender-specific differences in CBD effects when comparing this work with our previously published study. Unfortunately, we are unable to provide evidence for similar divergence in effects of citalopram since we previously did not evaluate memory effects of this compound (see also Uhernik et al. 2018). To summarize this, CBD showed sex-dependent affects on contextual fear memory extinction while citalopram did not affect either memory type. Also, CBD and citalopram inhibited generalized fear, and while we could show the CBD effect was sexually dimorphic, we did not have evidence from males to evaluate this for citalopram. Overall, a limitation of our sexual dimorphism findings is that male and female mice were studied in separate experiments done years apart; however, we used an identical experimental design in both studies, with the same strain of mice from the same supplier, identical housing, bedding, food, animal handling, environmental conditions, and diurnal cycle. Also, the CBD in each study was from the same source, given at the same dose, with identical vehicle solutions. The only major differences between the two studies was that the female mice in this study were 42-50 days old when the study began, whereas the male mice in Uhernik et al. were 24-25 days old, and, females froze considerably more than males in vehicle-treated groups, as would be expected based on well-established sexual differences (for example see Day and Stevenson 2020). Therefore, the combined gender differences in the effects of CBD on contextual and generalized memory suggest it could be useful for PTSD in women, but harmful for men. Clearly, more research into the sexually dimorphic effects of both CBD and SSRIs on fear memory acquisition and consolidation is needed to further resolve the appropriate use of either drug for treating disorders of memory and learning.

## Data Availability

Upon request

## Abbreviations

PTSD: Post traumatic stress disorder
SSRIs: Selective serotonin reuptake inhibitors
CBD: Cannabidiol
CIT: Citalopram
CS: Conditioning stimulus
US: Unconditional stimulus
NS: Not statistically significant
EX1: Extinction training day 1
IP: Intraperitoneally
ITI: Inter-trial interval
